# *De novo* designed peptides form a highly catalytic ordered nanoarchitecture on a graphite surface†

**DOI:** 10.1039/d2nr01507b

**Published:** 2022-05-26

**Authors:** Wei Luo, Hironaga Noguchi, Chen Chen, Yoshiki Nakamura, Chishu Homma, Oleksii Zozulia, Ivan V. Korendovych, Yuhei Hayamizu

**Affiliations:** Department of Materials Science and Engineering, School of Materials and Chemical Technology, Tokyo Institute of Technology Tokyo 152-8550 Japan hayamizu.y.aa@m.titech.ac.jp; Department of Chemistry, Syracuse University Syracuse New York 13244 USA

## Abstract

Here we demonstrate that short peptides, *de novo* designed from first principles, self-assemble on the surface of graphite to produce a highly robust and catalytic nanoarchitecture, which promotes peroxidation reactions with activities that rival those of natural enzymes in both single and multi-substrate reactions. These designable peptides recapitulate the symmetry of the underlying graphite surface and act as molecular scaffolds to immobilize hemin molecules on the electrode in a hierarchical self-assembly manner. The highly ordered and uniform hybrid graphite–peptide–hemin nanoarchitecture shows the highest faradaic efficiency of any hybrid electrode reported. Given the explosive growth of the types of chemical reactions promoted by self-assembled peptide materials, this new approach to creating complex electrocatalytic assemblies will yield highly efficient and practically applicable electrocatalysts.

## Abstract 2

*De novo* designed peptides self-assembled on a graphite surface to produce highly robust and active electrocatalysts and promote peroxidation reactions with activities that rival those of natural enzymes in both single and multi-substrate reactions.

## Graphical abstract

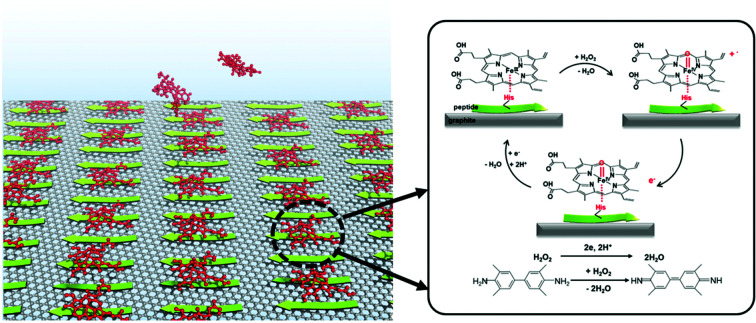

## Introduction

*De novo* designed short peptides have recently emerged to form nano-assemblies in solution and exhibited chemical reactions with activities that rival those of natural enzymes.^1^ The rapidly growing repertoire of the reactions promoted by these materials, often referred to as catalytic amyloids,^2^ their versatility in productive accommodation of various cofactors,^3^ their highly designable sequences, and their simple molecular structures that can be produced in large amounts at low cost offer an exciting possibility of creating highly efficient hybrid materials for different electrochemical applications. Yet, all the currently reported applications of these catalytic peptide amyloids were demonstrated in solution, and no attempts to incorporate these into complex assembled structures on solid surfaces were made.

Graphene, a single layer of graphite, has motivated much research due to its unique electrical and physicochemical properties, including electrical conductivity, large surface area, chemical robustness, and mechanical stability.^4^ For electrochemical applications, graphene can be functionalized by partial oxidation and immobilization of functional molecules on its surface.^5–7^ Simultaneously, peptide-functionalized graphene electrodes have been shown to have applications in biosensing.^8–12^ Moreover, graphitic surfaces have been used as templates for peptides to form highly ordered nanostructures,^9,11–17^ offering a unique possibility to act as a versatile platform for hierarchical self-assembly.^18,19^ Hybrid architectures with cofactors supported by self-assembled peptides on graphitic surfaces can be highly efficient and robust electrocatalysts for a variety of practical applications.^5,20–24^

We hypothesized that assembling catalytic peptides on a graphitic surface would fully harness the catalytic potential of peptide assemblies and create a highly functional nanoarchitecture capable of efficiently catalyzing practical chemical reactions. In a proof-of-concept study, we focused on peroxidation reactions due to their potential applications in various processes ranging from cancer therapy,^25^ wastewater purification,^26,27^ to polymer production.^28^ In particular, enhanced capability of accelerating the reduction of H_2_O_2_ is essential for constructing electrocatalysts for various peroxidation applications due to its role as an electron acceptor in most peroxidase reactions. Furthermore, peroxidase activity in natural enzymatic^29^ and model systems^30^ has been extensively studied, providing important activity and stability benchmarks.

**Fig. 1:**
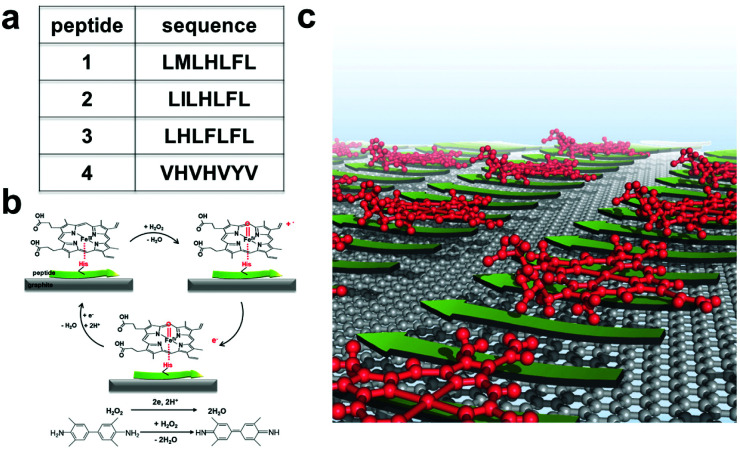
(a) Sequences of the four peptides studied. (b) The reactions catalyzed by the electrodes functionalized by the hybrid system of self-assembled peptides. (c) Schematic representation of the self-assembled hybrid interface consisting of peptides (green arrows) and hemin (red molecules) on a graphite surface.

To test our hypothesis, we employed several peptides from a recent report.^31^ These peptides were found to self-assemble in the presence of hemin into catalytic amyloids with high peroxidation activities in solution. Among them, we selected highly active (LMLHLFL and LILHLFL) and moderately active (LHLHLFL and VHVHVYV) peptides in this work (Fig. 1a). First, we investigated their ability to form highly ordered nanostructures on graphite surfaces, and then immobilized hemins on peptides to evaluate their catalytic activities by electrochemical measurements over H_2_O_2_. Additionally, we introduced 3,3′,5,5′-tetramethylbenzidine (TMB) to demonstrate the model of peroxidase reactions for multiple substrates and analyzed the efficiency during the electron transfer process (Fig. 1b). The robust structure, ease of production, and high peroxidation activities of our hybrid system make them notable interfaces for electrocatalysis.

## Results and discussion

**Fig. 2:**
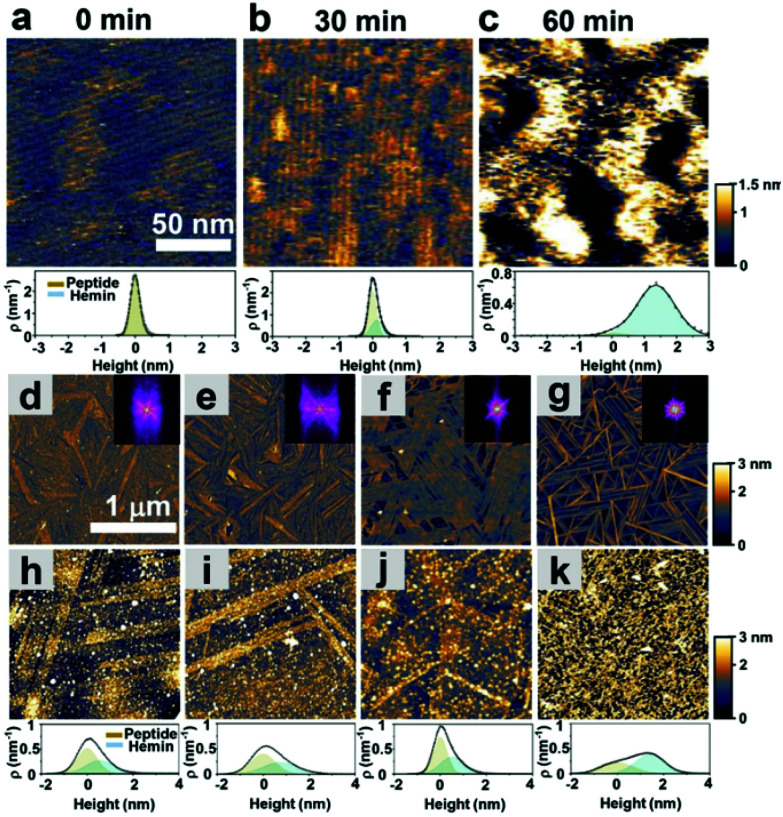
Self-assembly of catalytic peptides on a graphite surface and hemin adsorption on the self-assembled peptides. (a)–(c) *In situ* AFM height images and height distribution of hemin adsorbed on the self-assembled peptide 1 at various times. (d)–(g) *ex situ* AFM height images of the respective peptides of 1, 2, 3, and 4 assembled at a 1 μM concentration. The insets show the corresponding fast-Fourier transform images. (h)–(k) *ex situ* AFM height images (dry) and height distribution of the samples of 1, 2, 3, and 4 after immobilizing hemin. The fits show deconvolution of the contributions of the peptides and hemins.

As shown in Fig. 1c, we hypothesized that the peptides form long-range ordered structures on graphite surfaces in a self-assembly manner and support the immobilization of hemins on them. The ability of peptides 1–4 to self-assemble on graphite surfaces was tested first. In all cases, the peptides formed long-range ordered structures on the graphite surface as revealed by atomic force microscopy (AFM) studies under wet (Fig. 2a–c, Fig. S1–S3 and ESI section 1†) and dry conditions (Fig. 2d–g, Fig. S4 and ESI section 2†), respectively. We defined AFM measurements under wet and dry conditions as *in situ* AFM and *ex situ* AFM, respectively. All the peptides exhibited well-ordered linear structures with dissociation constants for graphite binding ranging from 125 to 294 nM (Fig. S5 and S6 and ESI section 3†). The images of fast Fourier transform corresponding to each height image show a clear six-fold symmetry indicating that the peptide self-assembly was templated by the graphite crystal lattice (insets of Fig. 2d–g), consistent with previous reports on peptide assembly on the graphite surface.^11,12,14–16,17,32^ The self-assembled structures formed by 1–4 exhibited high stability in rinsing experiments. After rinsing the surface with water and hydrogen peroxide solution, the original structures remained as shown by AFM (Fig. S7 and S8 and ESI section 4†). The stability is essential for being a molecular scaffold for functional hemin immobilization.

Having established the stability of the self-assembled peptides on the graphite surface, we studied their ability to immobilize hemin molecules. After incubating a 1 μM hemin solution on the self-assembled peptides for one hour, the surface morphology of peptide self-assembled structures was characterized by AFM (Fig. 2d–g). The concentration of the hemin solution was optimized to avoid the aggregation of hemin on the surface (Fig. S9†) and was used for all subsequent electrochemical experiments. AFM images show hemin molecules bound on each self-assembled peptide, where bright dots appeared on the linear structures of peptides (Fig. 2h–k). Height distributions of AFM images were fitted with Gaussian peaks to estimate the coverage and height of hemin molecules on the surface (ESI, sections 5 and 6†). For peptide 1, we performed an *in situ* AFM experiment monitoring the adsorption process of hemin on self-assembled structures formed by peptide 1 (Fig. 2a–c, Fig. S1†). Before the observation, peptide 1 was incubated on the graphite surface. At 0 minutes, right after hemins were injected into the solution, aligned nanowires of peptides in one direction were observed with a 5 nm separation (Fig. 2a and Fig. S1†). After 30 minutes, an adlayer formed on the peptide structures (Fig. 2b and c). The height increases are seen in the height histogram (Fig. 2a–c), indicating the successful binding of hemin molecules supported by the peptide assemblies.

**Fig. 3:**
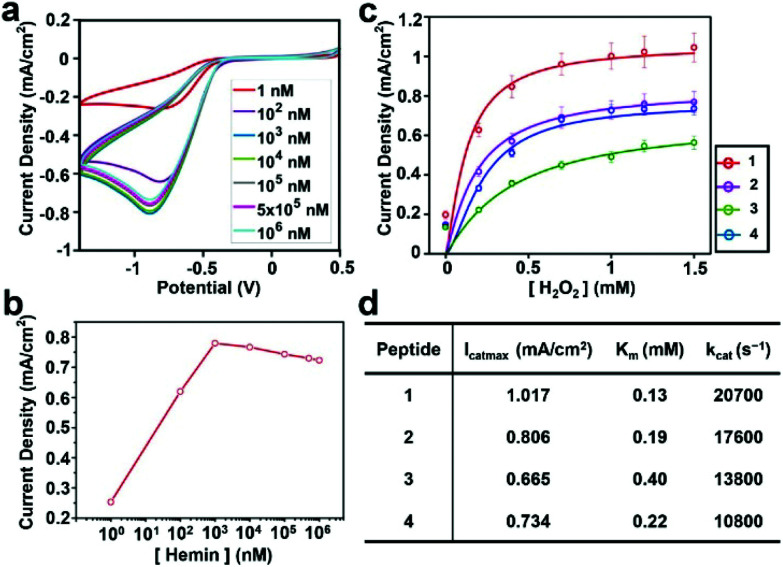
Electrochemical reduction of H_2_O_2_ with graphite–peptide–hemin hybrid electrodes. (a) Cyclic voltammograms at various concentrations of hemin. (b) The current density of the reduction peak *vs.* hemin concentration measured in 1 mM H_2_O_2_ solution. (c) Current density at the reduction peak with various concentrations of H_2_O_2_ with 1 μM hemin. Solid lines show fitting curves with the Michaelis–Menten model. (d) Summary of the kinetic parameters.

Next, we evaluated the ability of the assemblies to perform electrocatalysis. First, we analyzed the apparent kinetic parameters for the electrochemical peroxidation using H_2_O_2_ as a single substrate on a hybrid electrode formed by 4 and hemin. Cyclic voltammetry (CV) was performed with electrodes functionalized with self-assembled peptides prepared with hemin concentrations ranging from 1 nM to 1 mM (Fig. 3a). The peak at a potential of −0.75 V on the negative scan corresponds to H_2_O_2_ reduction for the peroxidase-like activity of the hybrid electrode. Consistent with the AFM data on hemin binding (Fig. S9†), the current density increases linearly until the hemin concentration reaches 1 μM and decreases at higher concentrations, presumably due to unproductive aggregations of hemins on the electrode surface making the catalytic sites inactive (Fig. 3b).

The current densities of each electrode at different H_2_O_2_ concentrations were measured to characterize their catalytic activity. The data were fitted with a Michaelis–Menten model (Fig. 3c), and the derived kinetic parameters are given in Fig. 3d. The details of the method to estimate these parameters are given in the Experimental section. All peptide–hemin hybrid electrodes showed higher peroxidase-like activity than the hemin-graphite electrode alone (Fig. S10†). The values obtained for the hybrid electrodes surpass those reported for artificial peroxidase electrodes (Table S1, ESI†).

**Fig. 4:**
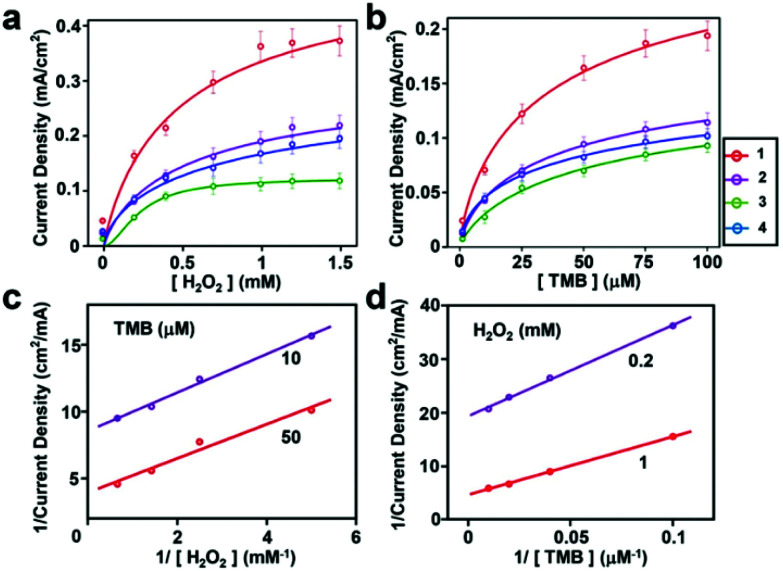
Kinetic assays of the hemin–peptide–graphite electrode. The current density was measured at a peptide concentration of 1 μM and a hemin concentration of 1 μM. (a) Current density at a TMB concentration of 375 μM with various H_2_O_2_ concentrations. (b) Current density at a H_2_O_2_ concentration of 5 mM with various TMB concentrations. (c) and (d) Double-reciprocal plots of the current density of hemin–peptide graphite electrodes at fixed concentrations of one substrate *versus* varying concentrations of the second substrate for H_2_O_2_ and TMB. 100% electron transfer efficiency was assumed. Solid lines show the fitting curves.

Encouraged by the catalytic efficiency of the peptide-modified hybrid electrodes in peroxidase activity using a single substrate, we studied their performance in a multi-substrate setting. Multi-substrate peroxidation is practically crucial as it offers an ability to utilize the oxidizing power of the high-valent iron species generated in this reaction for a broad range of applications. However, it is significantly more demanding as sufficient binding of multiple substrates to the catalyst stable under the reaction conditions is required for efficient turnover. We chose the commonly used substrate TMB to test for the peroxidation activity due to its ability to form colored oxidation products and previously reported data providing a benchmark for our results. We measured the catalytic activity of hemin–peptide–graphite electrodes over a range of H_2_O_2_ and TMB concentrations. Fits of the current density dependence on one substrate concentration were obtained for a fixed saturating concentration of the other substrate (Fig. 4a and b). The corresponding kinetic parameters are given in Table 1.

**Table 1:** Kinetic parameters of the peptides obtained from the electrochemical measurements

Peptide	*I*_cat_ max H_2_O_2_ (mA cm^−2^)	*K*_m_ H_2_O_2_ (mM)	*k*_cat_ H_2_O_2_ (s−1)	*I*_cat_ max TMB (mA cm−^2^)	*K*_m_ TMB (mM)	*k*_cat_ TMB (s^−1^)	*K*_S_ (s^−1^)
1	0.529	0.53	10 700	0.299	45.23	6000	2.44
2	0.336	1.02	7300	0.183	52.72	4000	1.47
3	0.126	0.23	2600	0.152	63.03	3200	1.52
4	0.318	0.62	4700	0.181	73.13	2700	1.48

Similar to the results obtained for a single substrate, the catalytic abilities of all the electrodes modified with peptides and hemin were much higher than those of electrodes modified with hemin only (Fig. S11 and S12†). The double reciprocal plots of current density *versus* one substrate concentration with a fixed concentration of the other substrate (Fig. 4c and d) are parallel to each other, in agreement with the ping-pong kinetic mechanism observed with horseradish peroxidase.^33^ Using this mechanism, the hemin–peptide–graphite electrode undergoes a process of first reacting with hydrogen peroxide to form a high valent species, which subsequently oxidizes the organic substrate. The kinetic parameters indicated that the peptide-modified electrode showed high catalytic performance in single- and multi-substrate modes. Although the direct comparison with other previously reported peroxidation electrocatalysts (Table S2, ESI†) is not straightforward due to differences in reaction conditions, to the best of our knowledge, the kinetic constants we observed are among the highest reported. Compared with natural enzymes, the modified hemin–peptide–graphite electrode exhibited excellent substrate affinity, high turnover rate, and a similar catalytic mechanism, and shows potential for applications as an artificial enzyme that can be manufactured easily. Interestingly, in all cases, the observed K_m_ values for the organic substrate were lower by approximately one order of magnitude for the peptide electrodes compared to the values for the bulk solution, indicative of the improved affinity for the substrate of the ordered assemblies.

We assumed that the electron transfer efficiency in the electrocatalytic reactions was 100% in the kinetic analyses reported above. However, practically, this number is often substantially lower due to energy loss as heat and/or reaction byproducts.^34^ We conducted chronoamperometric and UV-Vis measurements to evaluate the electrocatalytic process and derive the Faradaic Efficiency (FE) of the peptide-modified electrode (ESI, Section7†). All peptide-modified electrodes showed excellent FE values ranging from ∼50% for 4 to 70% for peptide 1 (Fig. S13†). Furthermore, we estimated the heterogeneous electron transfer rates (K_s_) of hybrid electrodes from CV experiments (ESI, Section 8).^35^ The 1-hemin-graphite assemblies show the largest *K*_s_ value of 2.44 s^−1^ among the peptides tested, consistent with the results of the kinetic experiments (Fig. S14 and Table S3 and S4†). Notably, the *K*_s_ value for the assemblies formed by 1 is comparable to the highest value reported to date in all the systems where heme is not in direct contact with a conducting surface.^36–40^ In addition, peptide 1 had a stable reaction over multiple cycles under cyclic voltammetry for both single and multi-substrate reactions with H_2_O_2_ and TMB (Fig. S15†).

## Conclusions

In conclusion, we have demonstrated that catalytic supramolecular peptide assemblies can be efficiently and selectively assembled on graphite electrodes. The resulting highly ordered nanoarchitectures promote peroxidation with kinetic parameters approaching the natural enzymes in the same reaction at a fraction of the cost. Unlike the existing methods for preparing electrocatalytic electrodes, this approach allows for crystal lattice-directed assembly, wherein the symmetry of graphite supports the formation of highly ordered peptide assemblies. We created self-assembled structures with variable substrate affinities and electrochemical properties, yet still strongly associated with the electrode. This finding suggests a previously inaccessible possibility of molecular level fine-tuning to manufacture complex hybrid electrodes with variable properties, including selective molecular recognition of multiple substrates. The ability of peptides to form catalytic heteromeric assemblies with synergistic properties further expands the possibilities of creating functional and biocompatible materials based on their designability of the sequence. The self-assembling peptides are remarkably stable and work under a wide range of conditions in different solvents. Given the rapidly growing number of reports showing that catalytic peptide assemblies promote a wide variety of other chemical transformations,^41^ we believe that this strategy will be widely applicable for creating efficient, inexpensive, and environmentally compatible electrochemical catalysts.

## Experimental

### Materials

Highly ordered pyrolytic graphite (HOPG, SPI supplies) was exfoliated using a mechanical exfoliation method and transferred onto a glass slip as a working electrode. Peptides were synthesized by manual fluorenylmethyloxy-carbonyl (Fmoc) solid-phase synthesis following a previously reported protocol.^42^ Peptide stock solutions were prepared by dissolving lyophilized peptides (purity ≥95%) with 30% acetone in DI water to reach a final concentration of 4 mM. The stock solutions were then diluted with deionized water to 5 μM–100 nM. Hemin powder was purchased from Fujifilm. Hemin stock solution was made by dissolving hemin powder in DMSO (pH 8) with subsequent dilution with deionized water to the desired concentration.

### Preparation of atomic force microscopy (AFM) samples

The preparation of the *in situ* AFM samples was performed in solution. All the *in situ* AFM images were captured after 1-hour incubation of peptides followed by the replacement of the solutions with DI water to stop the self-assembly. The *in situ* AFM images monitoring the hemin immobilization were captured after injecting hemin aqueous solution into the above solution to reach a final concentration of 1 μM.

In sample preparation for *ex situ* AFM measurements, freshly prepared HOPG electrodes were incubated with peptide solutions (100 μL at various concentrations) at room temperature for 1 hour in a humid chamber to prevent evaporation. After the incubation, the peptide solution was gently blown off with nitrogen gas, and the electrode was dried in a vacuum desiccator overnight to remove any remaining solution.

### Preparation of hybrid HOPG electrodes

For the hemin immobilization, we replaced the peptide solution with DI water after 1-hour incubation of peptide solutions on HOPG in a humid chamber at room temperature, and then replaced it with 40 μl of 1 μM hemin solution without drying up the samples. After waiting for 1 hour, the hemin solution was removed using a flow of nitrogen gas to produce a HOPG–peptide–hemin HOPG electrode.

### Morphological characterization by AFM

The morphology of peptides self-assembled or hemin-bound peptides on graphite was observed using an atomic force microscope (MFP-3D-SA, Asylum Research, Oxford Instruments) in air. The AFM instrument was equipped with a silicon cantilever (OMCL-AC160TS, Olympus, JP) with a resonance frequency of 300 ± 100 kHz and a spring constant of 26 N m^−1^. The scanning speed was around 1.5 Hz. The tip radius was 9 ± 2 nm. The high resolution *in situ* images were obtained by AFM in liquid mode with a silicon cantilever (Cypher, Asylum Research, Oxford Instruments) with a resonance frequency of 25 kHz and a spring constant of 0.1 N m^−1^. The scanning speed was around 2.5 Hz. The software Gwyddion (Czech Metrology Institute, CZ) was utilized for analyzing AFM images. For estimation of the hemin coverage, we converted the height information into a height histogram and fitted it with Gaussian functions to identify peaks. The peak at lower height was assigned to the peptide nanostructure, while the peaks at higher height were attributed to the bound hemin.

### Electrochemical characterization

Electrochemical measurements, including cyclic voltammetry (CV) and chronoamperometry, were performed using a Versastat3 from Princeton Applied Research with a three-electrode system. A platinum wire, Ag/AgCl electrode, and modified electrode were used as the counter electrode, reference electrode, and working electrode, respectively. The electrocatalytic reduction of H_2_O_2_ and the coupling catalytic reduction of H_2_O_2_ with the redox reaction of TMB were carried out in 0.1 M phosphate buffer (pH 7) solution with 10 successive cyclic voltammograms. The stock solution of H_2_O_2_ (1 mM) and TMB (1 mM) was diluted into desired concentrations. The ranges of scanning potential for the single substrate (H_2_O_2_) and multi-substrate (H_2_O_2_ and TMB) reactions were −1.4 to 0.5 V and 0 to 0.9 V, respectively, at a scan rate of 0.5 V s^−1^. Prior to all electrochemical experiments, the solutions were degassed with high purity nitrogen for 15 minutes, and a nitrogen atmosphere was maintained during the measurements.

Chronoamperometry measurements for TMB oxidation in the presence of H_2_O_2_ were performed at a potential of 0.32 V. UV-Vis absorption spectra were recorded using a NanoPhotometer (N60, IMPLEN, JP).

An electrochemical form of the Michaelis–Menten formalism was used to analyze the catalytic activity of the hybrid electrode.^43^
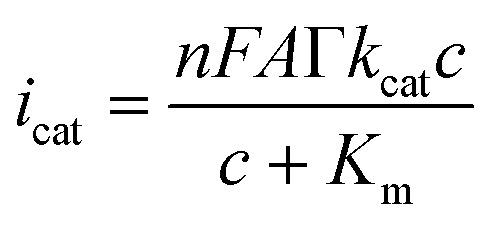
where *i*_cat_ is the current of the voltage position corresponding to the reduction peak (H_2_O_2_), or the oxidation peak (TMB); *n* is the number of transferred electrons per one reaction; *F* is the Faraday constant; *A* is the area of the electrode; *Γ* is the number of enzyme molecules per unit area of the electrode; *k*_cat_ is the turnover rate of the reaction; *c* is the bulk concentration of the substrate; and *K*_m_ is the apparent Michaelis constant.

To investigate the mechanism of the enzymatic reaction, multi-substrate measurements were carried out under standard reaction conditions as described (Fig. 4) by varying the concentration of one substrate at a fixed concentration of the other substrate. For the subsequent analysis, a double reciprocal plot was utilized to analyze the catalytic process.

## Conflicts of interest

There are no conflicts to declare.

## Supplementary Material

NR-014-D2NR01507B-s001

## References

[cit1] Rufo C. M., Moroz Y. S., Moroz O. V., Stohr J., Smith T. A., Hu X., DeGrado W. F., Korendovych I. V. (2014). Nat. Chem..

[cit2] Makhlynets O. V., Gosavi P. M., Korendovych I. V. (2016). Angew. Chem., Int. Ed..

[cit3] Zozulia O., Korendovych I. V. (2020). Angew. Chem., Int. Ed..

[cit4] Novoselov K. S., Geim A. K., Morozov S. V., Jiang D., Zhang Y., Dubonos S. V., Grigorieva I. V., Firsov A. A. (2004). Science.

[cit5] Jiang S., Cheng R., Wang X., Xue T., Liu Y., Nel A., Huang Y., Duan X. (2013). Nat. Commun..

[cit6] Wang S., Cazelles R., Liao W. C., Vázquez-González M., Zoabi A., Abu-Reziq R., Willner I. (2017). Nano Lett..

[cit7] Xuan X., Yoon H. S., Park J. Y. (2018). Biosens. Bioelectron..

[cit8] Khatayevich D., Page T., Gresswell C., Hayamizu Y., Grady W., Sarikaya M. (2014). Small.

[cit9] Page T. R., Hayamizu Y., So C. R., Sarikaya M. (2012). Biosens. Bioelectron..

[cit10] Islam A. E., Crasto C. M., Crasto C. M., Kim S. S., Rao R. S., Maruyama B., Drummy L. F. (2020). ACS Appl. Nano Mater..

[cit11] Hayamizu Y., So C. R., Dag S., Page T. R., Starkebaum D., Sarikaya M. (2016). Sci. Rep..

[cit12] Mustata G. M., Kim Y. H., Zhang J., Degrado W. F., Grigoryan G., Wanunu M. (2016). Biophys. J..

[cit13] Katoch J., Kim S. N., Kuang Z., Farmer B. L., Naik R. R., Tatulian S. A., Ishigami M. (2012). Nano Lett..

[cit14] Brown C. L., Aksay I. A., Saville D. A., Hecht M. H. (2002). J. Am. Chem. Soc..

[cit15] Zhang F., Du H., Zhang Z., Ji L., Li H., Tang L., Wang H., Fan C., Xu H., Zhang Y. (2006). Angew. Chem., Int. Ed..

[cit16] So C. R., Hayamizu Y., Yazici H., Gresswell C., Khatayevich D., Tamerler C., Sarikaya M. (2012). ACS Nano.

[cit17] Li P., Sakuma K., Tsuchiya S., Sun L., Hayamizu Y. (2019). ACS Appl. Mater. Interfaces.

[cit18] Ariga K., Jia X., Song J., Hill J. P., Leong D. T., Jia Y., Li J. (2020). Angew. Chem., Int. Ed..

[cit19] Cortez M. L., De Matteis N., Ceolín M., Knoll W., Battaglini F., Azzaroni O. (2014). Phys. Chem. Chem. Phys..

[cit20] Park D. H., Vieille C., Zeikus J. G. (2003). Appl. Biochem. Biotechnol..

[cit21] Leech D., Kavanagh P., Schuhmann W. (2012). Electrochim. Acta.

[cit22] Luong J. H. T., Glennon J. D., Gedanken A., Vashist S. K. (2016). Microchim. Acta.

[cit23] Ryu W. H., Gittleson F. S., Thomsen J. M., Li J., Schwab M. J., Brudvig G. W., Taylor A. D. (2016). Nat. Commun..

[cit24] Grieshaber D., MacKenzie R., Vörös J., Reimhult E. (2008). Sensors.

[cit25] Sadiq M., Pang L., Johnson M., Venkatachalem S., Wang D. (2020). Biosensors.

[cit26] Miller D. J., Dreyer D. R., Bielawski C. W., Paul D. R., Freeman B. D. (2017). Angew. Chem., Int. Ed..

[cit27] Wang J., Hu J., Hu S., Gao G., Song Y. (2020). Sensors.

[cit28] Ren Y., Wang Z. Y., Wei X., Xu L., Gul R. M., Huang S. S., Xu J. Z., Li Z. M. (2020). ACS Appl. Bio Mater..

[cit29] Cosnier S. (1999). Biosens. Bioelectron..

[cit30] Mugesh G., Singh H. B. (2000). Chem. Soc. Rev..

[cit31] Zozulia O., Marshall L. R., Kim I., Kohn E. M., Korendovych I. V. (2021). Chem. – Eur. J..

[cit32] Sun L., Narimatsu T., Tsuchiya S., Tanaka T., Li P., Hayamizu Y. (2016). RSC Adv..

[cit33] Porter D. J., Bright H. J. (1983). J. Biol. Chem..

[cit34] Jones J. E., Hansen L. D., Jones S. E., Shelton D. S., Thorne J. M. (1995). J. Phys. Chem..

[cit35] Laviron E. (1979). J. Electroanal. Chem. Interfacial Electrochem..

[cit36] Li Q., Zhang Y., Li P., Xue H., Jia N. (2019). Mikrochim. Acta.

[cit37] Malecka K., Ferapontova E. E. (2021). ACS Appl. Mater. Interfaces.

[cit38] Samourgkanidis G., Nikolaou P., Gkovosdis-Louvaris A., Sakellis E., Blana I. M., Topoglidis E. (2018). Coatings.

[cit39] Matheus M. M., de Groot T., Wonders A. H., Koper M. T. M. (2005). J. Am. Chem. Soc..

[cit40] Feng J.-J., Li Z.-H., Li Y.-F., Wang A.-J., Zhang P.-P. (2011). Microchim. Acta.

[cit41] Zozulia O., Dolan M. A., Korendovych I. V. (2018). Chem. Soc. Rev..

[cit42] Korendovych I. V., Kim Y. H., Ryan A. H., Lear J. D., DeGrado W. F., Shandler S. J. (2010). Org. Lett..

[cit43] Sucheta A., Cammack R., Weiner J., Armstrong F. A. (1993). Biochemistry.

