# Supplementary material for: De novo designed peptides form a highly catalytic ordered nanoarchitecture on a graphite surface
Source: Nanoscale. 2022 May 26;14(23):8326–31. doi: 10.1039/d2nr01507b (PMC9202597; doi:10.1039/d2nr01507b)
Supplement: NR-014-D2NR01507B-s001 [file NR-014-D2NR01507B-s001.pdf]

Electronic Supplementary Material (ESI) for Nanoscale.
This journal is © The Royal Society of Chemistry 2022

Electronic Supplementary Information

De novo designed peptides form highly catalytic ordered nanoarchitechture on graphite surface

Wei Luo, [a] *Hironaga Noguchi,* [a] *Chen Chen,* [a] *Yoshiki Nakamura,* [a] *Chishu Homma,* [a] *Oleksii Zozulia,* [b] *Ivan V. Korendovych,* [b] *Yuhei Hayamizu** [a]

[a] *Department of Materials Science and Engineering, School of Materials and Chemical Technology, Tokyo Institute of Technology, Tokyo 152-8550, Japan.*

[b] *Department of Chemistry, Syracuse University, Syracuse, New York 13244, USA*

Email: hayamizu.y.aa@m.titech.ac.jp

Table of Contents

1. Peptide self-assembled structures observed by in -situ AFM. Pages 3-6

2. Peptide self-assembled structures observed by ex-situ AFM. Pages 7-9

3. Estimation of peptide height and coverage from ex-situ AFM images. Pages 10-11

4. Stability of self-assembled structures. Pages 12-13

5. Binding of hemin to self-assembled peptides 4. Pages 14-16

6. Calculation of hemin density on self-assembled structure. Pages 16-17

7. Kinetic parameters of catalytic reactions. Pages 17-22

8. Electron transfer performance of the fabricated electrodes. Pages 23-25

9. References. Pages 26-27

1. Peptide self-assembled structures observed by in -situ AFM.

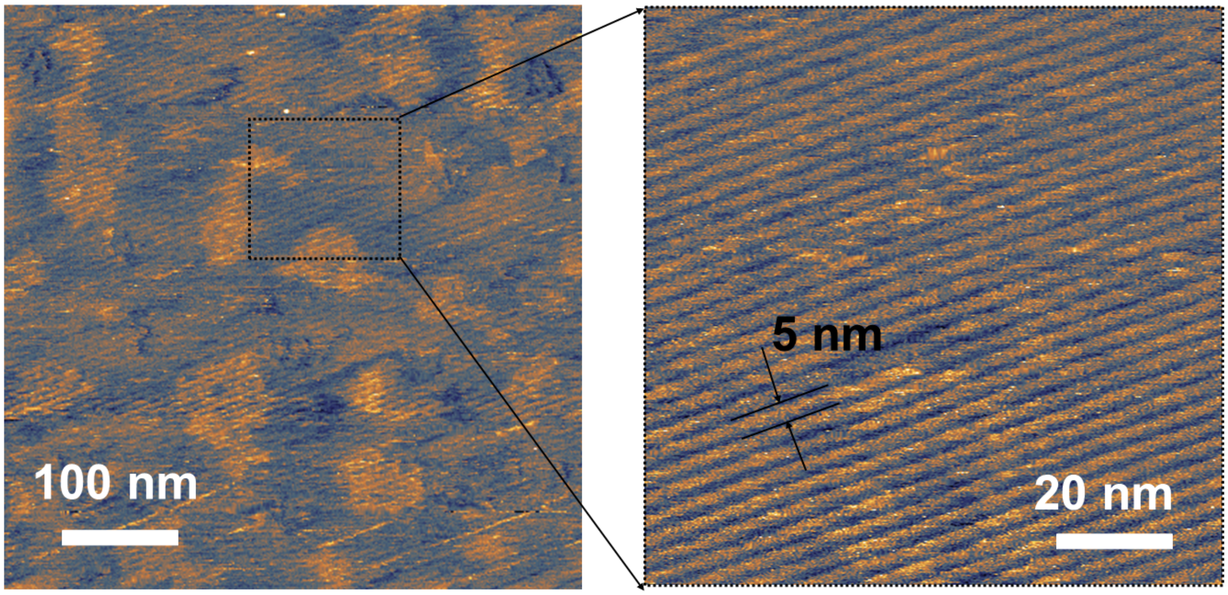

Figure S1. In-situ AFM height images of peptide assemblies formed by 1 μM aqueous solution of peptide **1**.

In this manuscript, AFM images measured under wet conditions were named “in-situ AFM image”, while AFM images measured under dry conditions were named “ex-situ AFM image”. In-situ AFM was utilized to observe the self-assembled structure at a high resolution (Fig. S1). The in-situ AFM (Cypher, Asylum Research, Oxford Instruments) was performed under DI water. Before the measurement, the graphite surface was incubated with 1-μM solution of peptide **1** for 1 hour, and the peptide solution was replaced with DI water. The AFM cantilever for the in-situ AFM was BL-AC40TS-C2, Biolever mini, Olympus. The high-resolution AFM image shows clear nanowire structures of peptide **1**, which are uniformly assembled on a graphite surface with a periodicity distance of 5 nm (Fig. S1). The length of the peptides with 7 amino acids in this work

is 2.5 nm. The 5-nm distance corresponds to the length of two peptides. These uniform structures can be observed in in-situ AFM imaging, but it is not possible in the case of ex-situ AFM measurement. The self-assembled structures of peptides can be denatured during the drying process. It is worth noting that the coverage and domain size of the self-assembled structures can be maintained even after the drying process.

Figure S2 depicted a model of our peptides on a graphite surface. Although the actual conformations of the peptides and hemins on the surface are unknown, we made the schematic to understand the size scale of each molecule for our eyes.

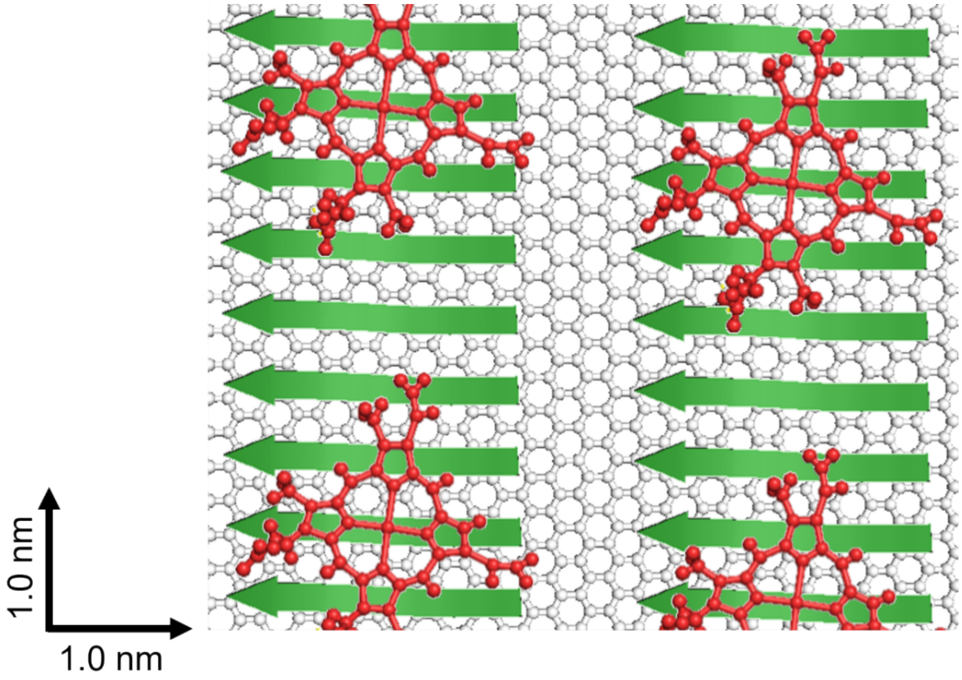

Figure S2. Schematic representation of the self-assembled hybrid interface consisting of peptides (green arrows) and hemins (red molecules) on a graphite surface.

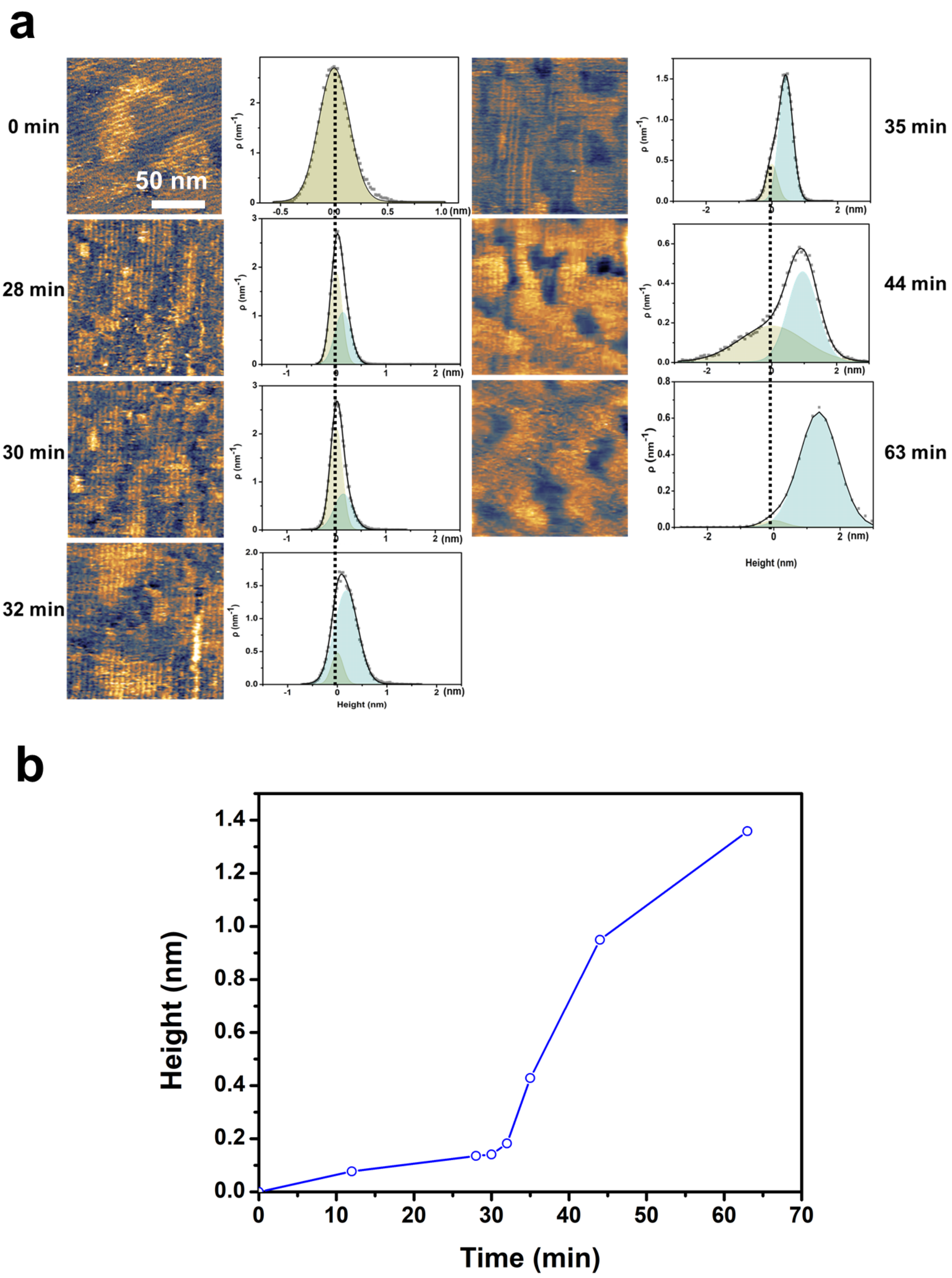

a
0 min
28 min
30 min
32 min
35 min
44 min
63 min
50 nm
ρ (nm-1)
Height (nm)
b
Height (nm)
Time (min)

Figure S3. (a) In-situ AFM images and the corresponding height histogram of hemin adsorbed on LMLHLFL at different time points. (Concentration: LMLHLFL, 1 μM; hemin, 1 μM.) (b) Height of adsorbed layer as a function of incubation time in Milli-Q water derived from in-situ AFM height images.

Fig. S3a shows a series of in-situ AFM images monitoring the binding of hemin to the self-assembled peptides at different time points after the injection of 1 μM hemin. After 1-hour incubation of peptides (1-μM aqueous solution), these images were taken on the surface. As shown in the height histogram obtained from the AFM height image, the height of the structures on the surface increases with time. The increase of the height as a function of time is plotted in Fig. S3b, showing that the height increased drastically after 30 mins.

2. Peptide self-assembled structures observed by ex-situ AFM.

The concentration dependence of the peptide self-assembly was examined by ex-situ AFM. The Ex-situ AFM images were taken after drying the samples. The AFM images have a size of 2 x 2 μm^2 to observe the uniformness and coverage of the structures. As shown in Fig. S4, these peptides formed long-range ordered structures on graphite surfaces, with linear domains aligning in each direction. The directions showed a six-fold symmetry similar to the graphite crystal lattices as recognized by FFT (Fast-Fourie Transform) analysis (insets in the AFM images, Fig. S4). Small dots observed in the 100-nM AFM images correspond to the aggregations of peptide molecules since there are insufficient numbers of peptides on the surface to form their long-range ordered structures.

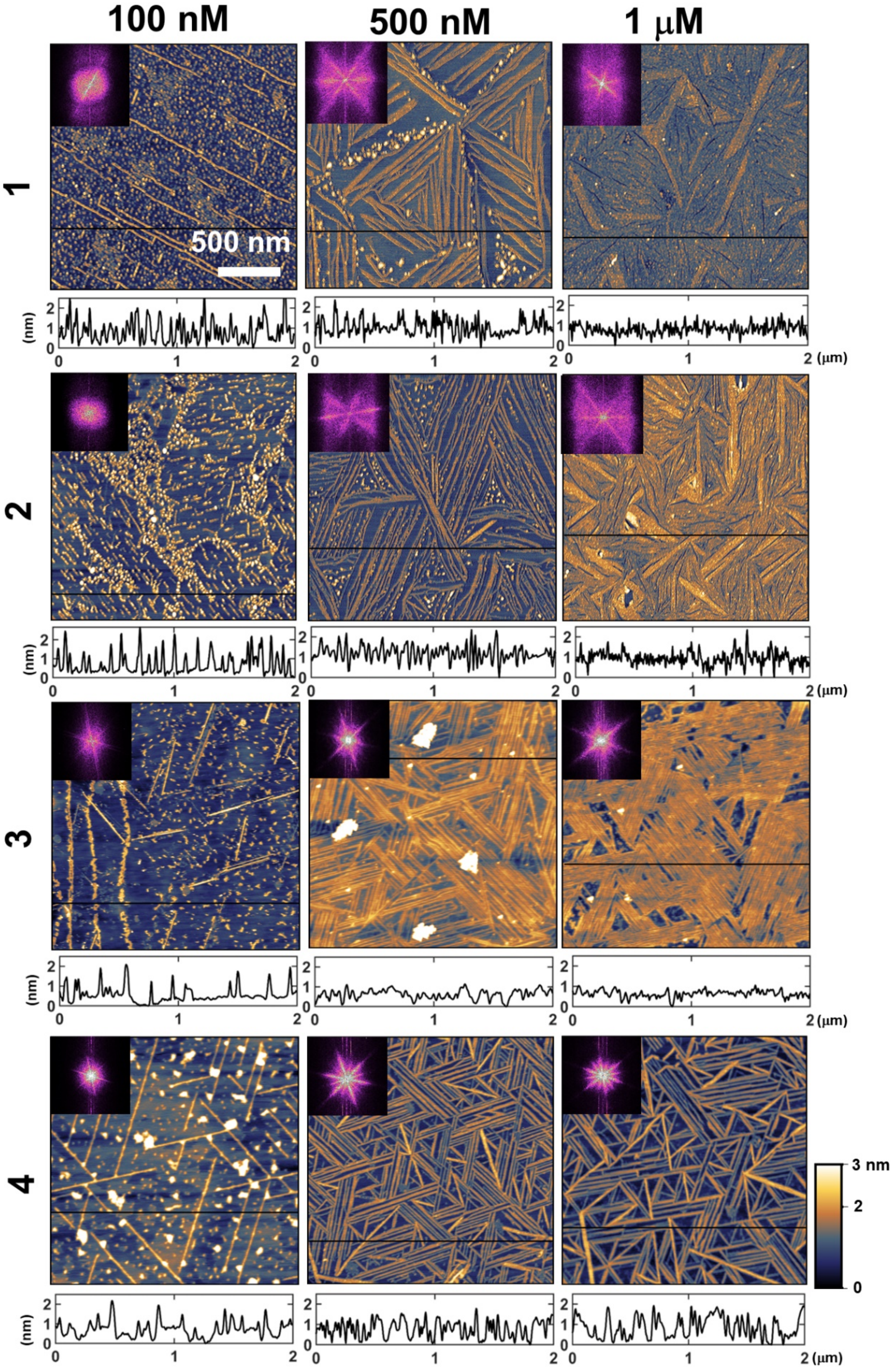

100 nM
500 nM
1 μM
1
2
3
4
500 nm
(nm)
(μm)
3 nm
2
0

Figure S4. Ex-situ AFM height images of peptide assemblies at various concentrations in the absence of hemin. The insets show the corresponding Fast-Fourier transform (FFT) images. The numbers on the left indicate the kinds of peptides. Each column shows AFM images at respective concentrations.

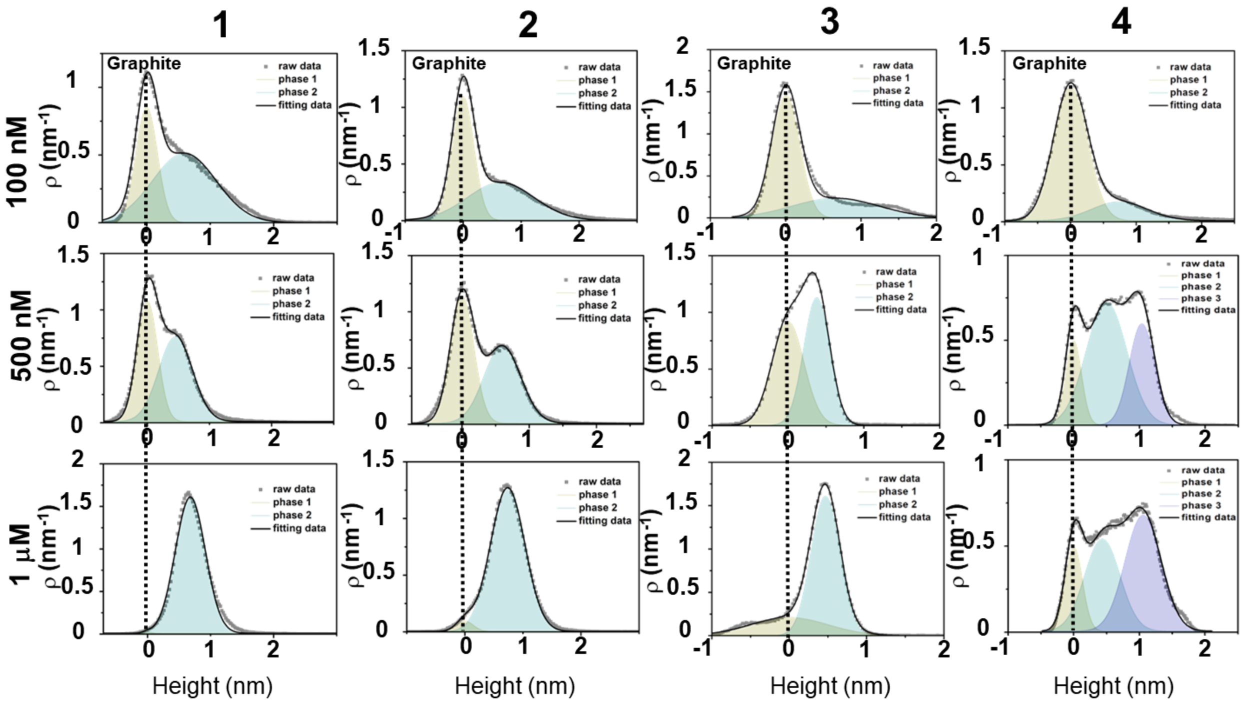

Figure S5. Height distribution of four peptides in different concentrations, fitting to Gaussian function model.

3. Estimation of peptide height and coverage from ex-situ AFM images.

To estimate the peptide coverage, we converted the height images (Fig. S4) into height histograms (Fig. S5) and fitted them with Gaussian functions to identify peaks. The peak at lower height was assigned to the bare surface of graphite, while the peaks at higher height were attributed to the peptide self-assembled layer. The peak position indicates the thickness of the peptide layer, varying in the range of 0.5-1.2 nm depending on the concentrations and the kind of peptides for the self-assembly (Fig. S5). Utilizing the area of each peak, we estimated the coverage of peptides on the surface.[1]

We found that peptide **4** (VHVHVYV) showed three peaks attributed to the bare graphite surface and two phases of peptide assembled structures on the graphite surface. Each peptide phase has a height of 0.5 and 1.0 nm, respectively. The doubled thickness of another phase indicates that the phase with the 1.0-nm thickness can be a bilayer of the phase of 0.5-nm thickness. Interestingly, the bilayer formation of peptide **4** can be correlated to multiple valines in the sequence, whereas the other peptides with multiple leucines form monolayer-like assemblies.

As increasing the concentration of peptide solution, the coverage of peptides increases. The coverages of peptides versus concentrations were plotted and fitted with the Langmuir isotherm model to estimate the peptide binding affinity to graphite (Fig. S6). The dissociation constants K_d of **1**, **2**, **3,** and **4** were estimated to be 294, 125, 208, 275 nM, respectively. The graphite surface was fully saturated at peptide concentrations of more than 1 μM. Thus, we utilized this concentration for the rest of the experiments.

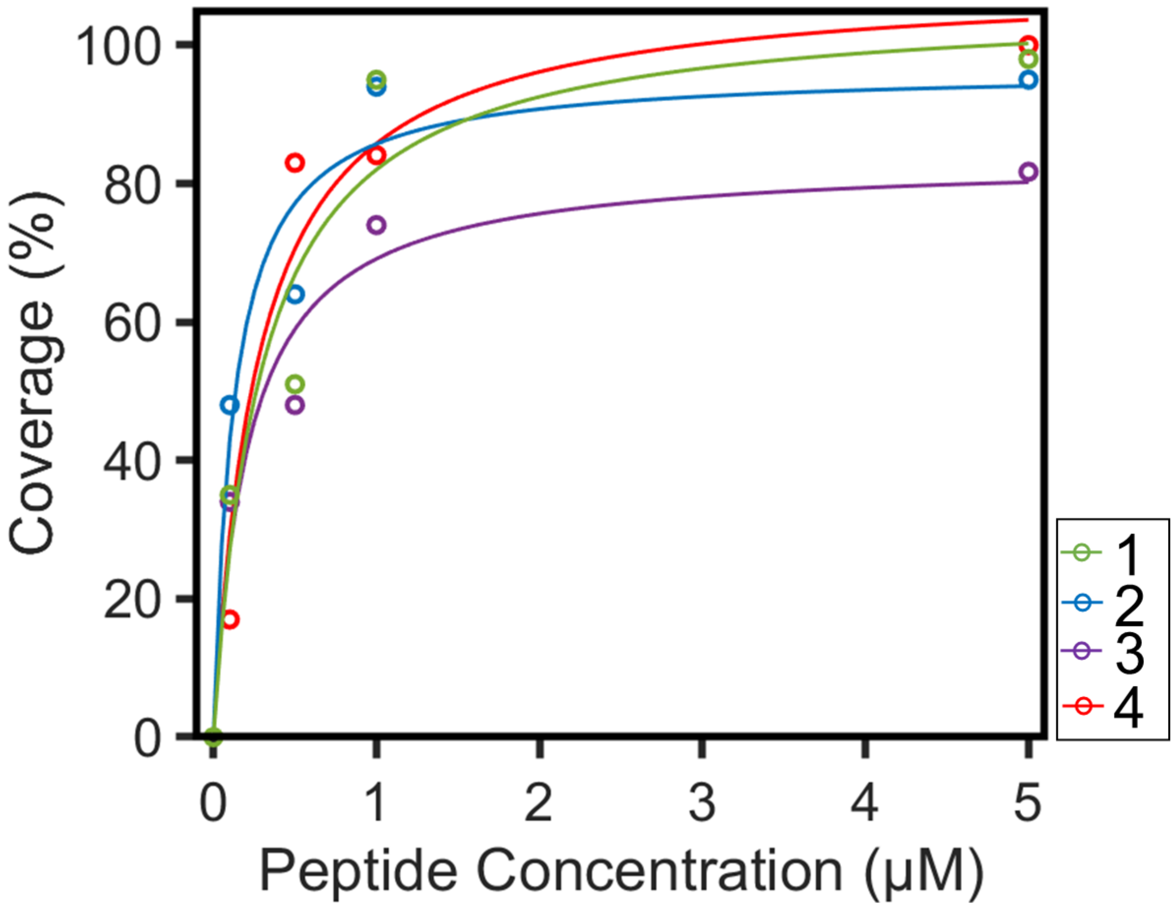

Figure S6. Coverages of peptides on graphite with different concentrations estimated from ex-situ AFM images in Fig. S4. Solid lines show fitting curves to the Langmuir model.

4. Stability of self-assembled structures.

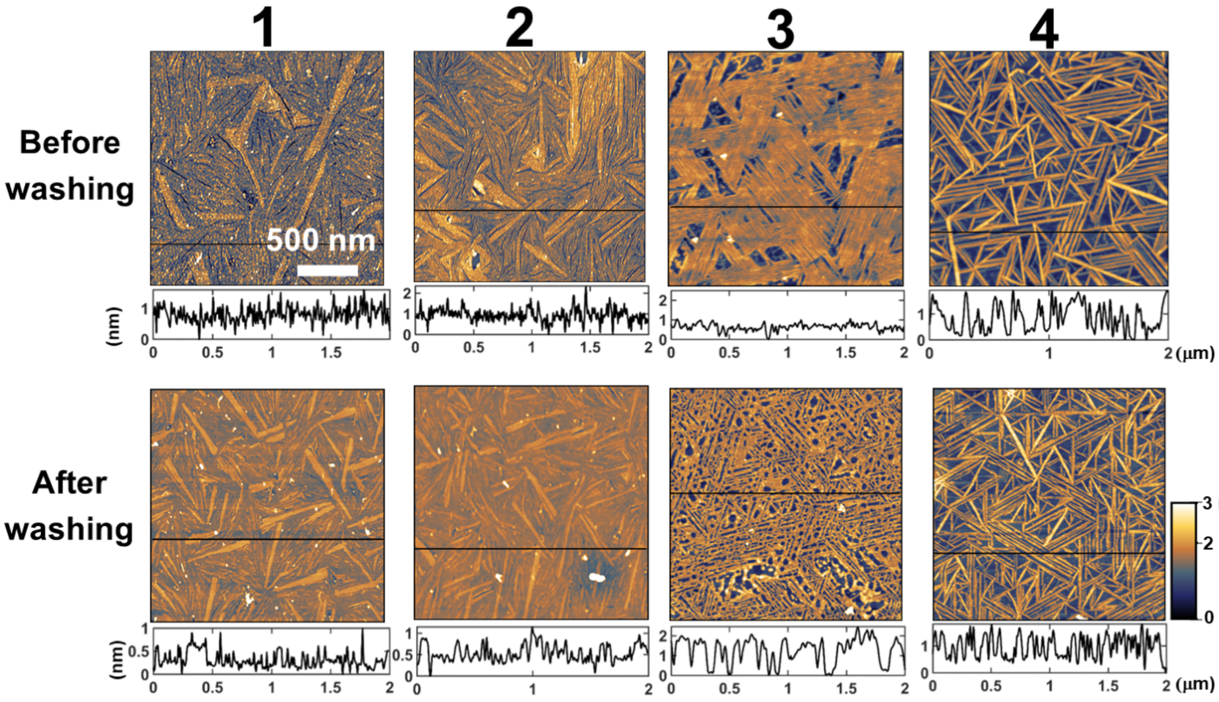

Figure S7. Ex-situ AFM images showing the stability of peptide self-assembled structures against water washing. The numbers on the top indicate the kinds of peptides.

The stability of the self-assembled structure of peptides was characterized by the following method. After one-hour incubation of peptide solutions on a graphite surface, the self-assembled structures were immediately washed with 100 μL of Milli-Q water. We replaced the incubation solution with fresh Milli-Q water every 30 seconds and repeated the washing process five times. After the fifth wash, the excess water was blown off by nitrogen gas, and the samples were dried overnight. Then, morphologies of the self-assembled structures on graphite were observed by ex-situ AFM. The coverage of peptides on graphite did not change after the washing process, proving

the outstanding stability of the self-assembled structures under Milli-Q water. The ordered structures formed by the peptides were also unaffected by washing with H_2O_2 (Fig. S8). In the H_2O_2 case, we duplicated the above washing process except for using H_2O_2 instead of Milli-Q water.

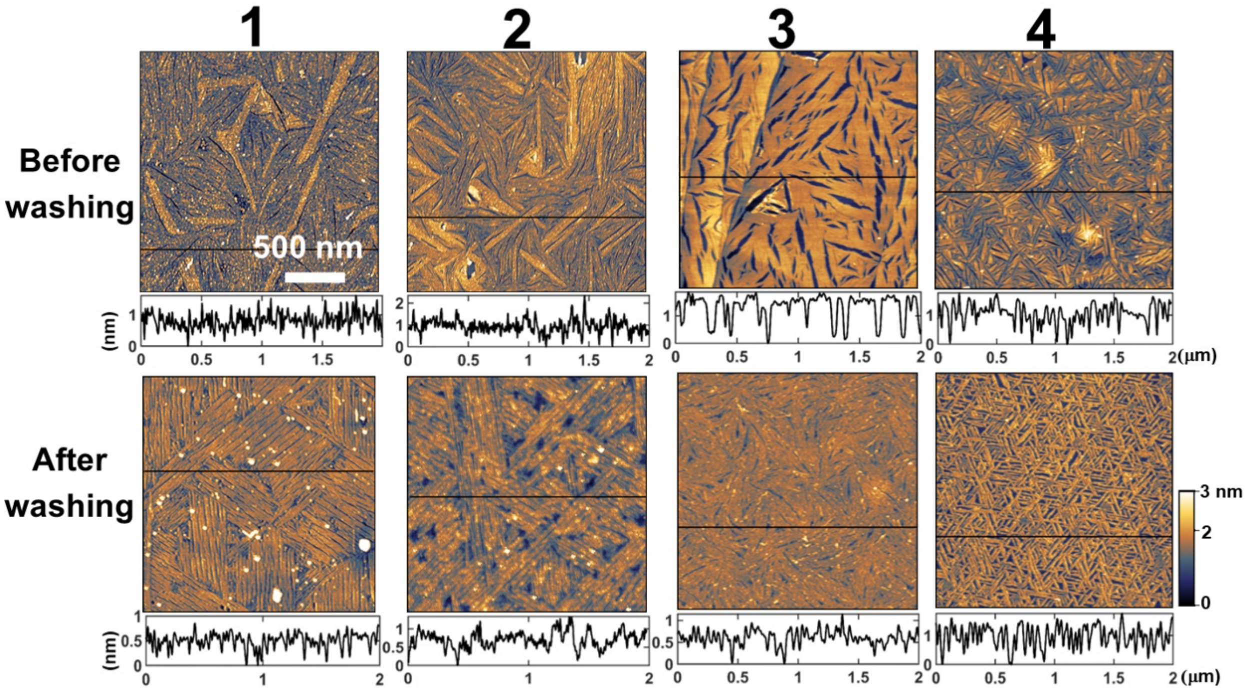

Figure S8. Ex-situ AFM images showing the stability of the self-assembled structures after washing with H_2O_2. The AFM images on the top were taken before the washing process, and the ones on the bottom were after the washing process.

5. Binding of hemin to self-assembled peptides 4.

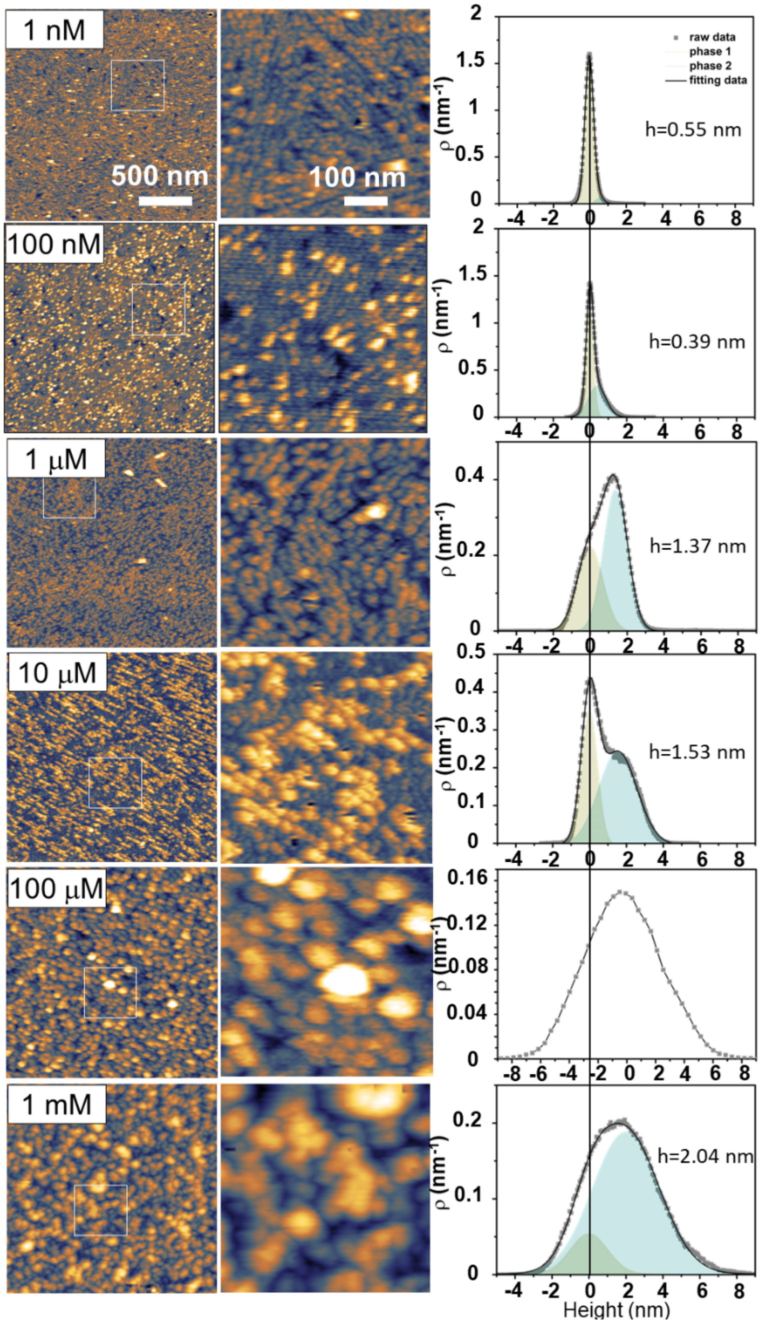

Figure S9. Ex-situ AFM images showing surface morphology and height profile of peptide-hemin on graphite surfaces. Each sample was prepared with different hemin solutions concentrations to search for a good condition of the hemin immobilization on the self-assembled peptides.

To study the effect of hemin concentration on their surface coverage, the stock solution of hemin was diluted to desired concentrations and then incubated on the top of self-assembled peptide structures for 1 hour. After the incubation, the hemin solution was blown off by nitrogen gas. Then, the morphology was observed by ex-situ AFM under dry conditions. Fig. S9 shows the AFM results and the corresponding height histograms. As the concentration of hemins increases, we can find an increasing number of bright spots in the height images. The histograms also show that the coverage and thickness of the hemins immobilized on the peptides increase as increasing the hemin concentration. Interestingly, there are linear structures under the bright spots in the case of the low concentration samples (less than 10 μM). It indicates that the linear structures of self-assembled peptides were preserved even after the hemin immobilization. In the samples with higher concentrations, we can not see these linear structures, probably due to the high coverage of the hemins on the surface. The samples with hemin concentrations of 1 and 100 nM showed that the thickness of bright spots was around 0.5 nm. It agrees with the previous observation of hemins on the graphene surface, demonstrating the monolayer thickness of hemins laying down parallel to the surface.[2] However, the thickness became larger in the samples with higher concentrations than 1 μM. The increasing thickness of the hemins in the histogram (Fig. S9) implies the aggregation or standing-up of hemins on the surface. Especially in the samples with higher concentrations of hemin than 10 mM, the lateral size of the bright spots got larger, indicating the aggregations of hemins on the surface. We utilized the hemin concentration of 1 μM in the electrochemical measurements as shown in Fig. 3b, plotting the current density of the reduction peak vs. hemin concentration measured in 1-mM H_2O_2 solution. We concluded from both AFM and electrochemical measurements that the 1-μM sample had a good balance between the high overage and less aggregation of hemins immobilized on the surface. Note that Fig. S3 also provides the

information of the increasing hemin thickness under the in-situ AFM observation. The thickness drastically increased after 30 minutes, implying the aggregation or standing-up of hemins on the self-assembled peptides (Fig. S3b).

6. Calculation of hemin density on the self-assembled structure.

The coverages of hemin on the self-assembled structure were calculated by analyzing the area of the higher peak of each plot (Fig. 2). The coverages of hemins with the peptide 1, 2, 3, 4 were 0.61, 0.43, 0.41, and 0.44, respectively. Based on the derived coverage of hemin, we estimated the surface density of hemin, Γ_{hemin}, by the following equation.

$$\Gamma_{hemin} = \frac{\theta_s}{A\theta_{hemin}} ,$$

where θ_s is the area of hemin on the surface estimated by an AFM image, θ_{hemin} is the area of a single hemin molecule (1.44 nm^2), A is the total area of the AFM image. We utilized 1.44 nm^2 for the θ_{hemin} in this work by assuming the conformations of hemins laying down parallel to the surface with the self-assembled peptides. As we discussed in the previous section, the thickness of the hemins in the samples prepared with 1 μM hemins was 1.4 nm, suggesting their aggregations or standing up. Due to the limitation of the spatial resolution of the AFM, we cannot determine the conformation or orientation of individual hemin molecules on the surfaces. Thus, for a rough estimation of kinetic parameters, we assume the simplest orientation of hemins as they are laying down parallel to the surface. It is reasonable when the hemin concentration was less than 1 μM in

our system since the samples with higher hemin concentrations gave much thicker hemins on the surface, clearly suggesting the aggregations of hemins.

7. Kinetic parameters of catalytic reactions.

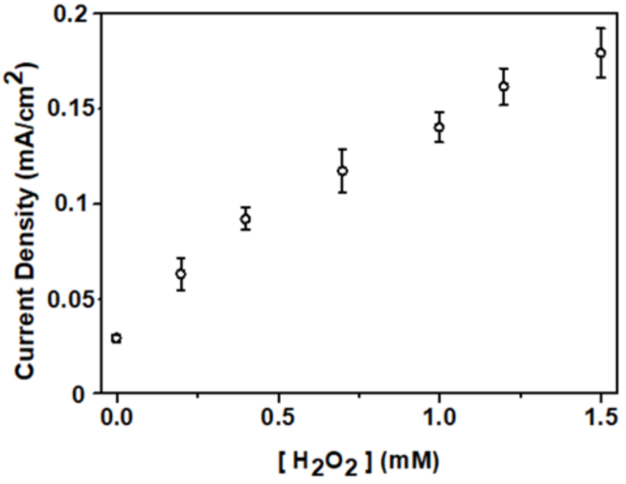

Figure S10. Current density at the reduction peak with various concentrations of H_2O_2 for the 1-μM hemin modified electrode.

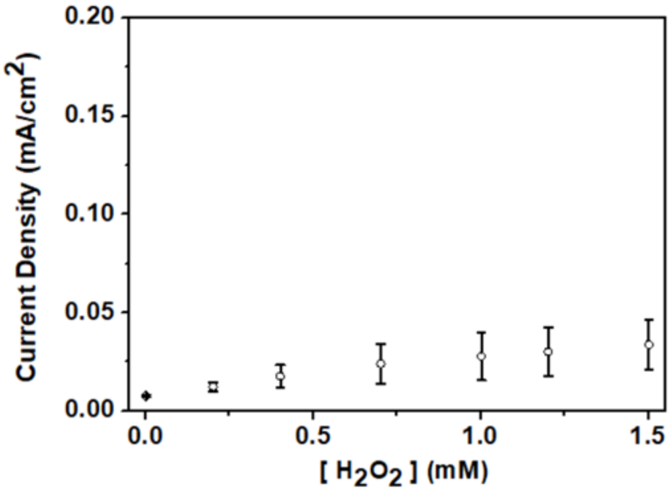

Figure S11. Current density at the TMB concentration of 375 μM with various H_2O_2 concentrations for the 1-μM hemin modified electrode.

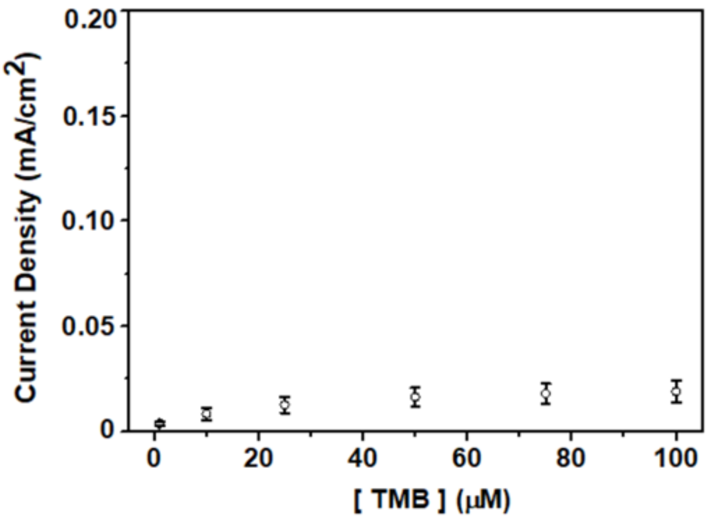

Figure S12. Current density at the H_2O_2 concentration of 5 mM with various TMB concentrations for the 1-μM hemin modified electrode.

Table S1. Summary of kinetic parameters reported in previous works demonstrating peroxidation electrocatalysts using H_2O_2 as a single substrate.

Catalyst	Vmax [*10^{-8} M s^{-1}]	K_m [mM]	k_{cat} [s^{-1}]	refs
GQDs-PH	0.211	1.09		3
GQDs-BA	2.67	1.12		
GQDs-BrPE	10.55	7.94		
GQDs	1.24	1.17		
GO-COOH	3.85	3.99		4
CQDs	30.61	26.77		5
GQDs	2.62	0.49		6
$C_{60}[C(COOH)_2]_2$	4.01	24.58		7
Carbon	2.07	49.8		8
Fe_3O_4 MNPs	9.87	154	85810	9
Hemin-micelle (PEG-b-P4VP)	52.62	2.17		10
H@M (MIL–101(Al)–NH_2)	8.98	10.9		11
H15	8	3.66	(1.147 ± 0.045)*10^{-4}	12
SWCNT	0.98	1.47		13
Hemin-SWCNT	4.79	0.08		
Cu–hemin MOFs	116	2.18		14
**4**-hemin	76.20	0.22	10800	This work
**3**-hemin	69.08	0.40	13800	
**2**-hemin	83.68	0.19	17600	
**1**-hemin	105.61	0.13	20700	

Table S2. Summary of kinetic parameters reported in previous works demonstrating peroxidation electrocatalysts using H_2O_2 and TMB.

Catalyst	Vmax [$*10^{-8}$M s^{-1}]	K_m [mM]	k_{cat} [s^{-1}]	refs
GO-COOH	3.45	0.0237		4
Fe_3O_4 MNPs	3.44	0.098	1.812*106	9
HRP	10	0.434	2.4*105	
H@M (MIL–101(Al)–NH_2)	6.07	0.068		11
H15 (histidine)		0.188	(1.322 ± 0.05)*10−4 s−1	12
(Fe−P)n-MOF		0.63		15
$CoFe_2O_4$	2.90	0.387		16
AHMT-Pd	850	0.03		17
V_2O_5 nanozymes	1850	0.738		18
NS-CQDs	0.1670	0.387		19
**4**-hemin	18.85	0.07313	2700	This work
**3**-hemin	15.79	0.06303	3200	
**2**-hemin	19.01	0.05272	4000	
**1**-hemin	30.99	0.04523	6000	

For easy comparison with other works, the current density at the voltage corresponding to the characteristic peak in the cyclic voltammogram was converted into the initial reaction rate v.

$$v = \frac{i_{cat}}{FN_A n_{cat}} \; ,$$

where i_{cat} is the current density of the voltage corresponding to the reduction peak (H_2O_2), or oxidation peak (TMB); F is the Faraday constant; N_A is the Avogadro constant; n_{cat} is the number of transferred electrons per one reaction.

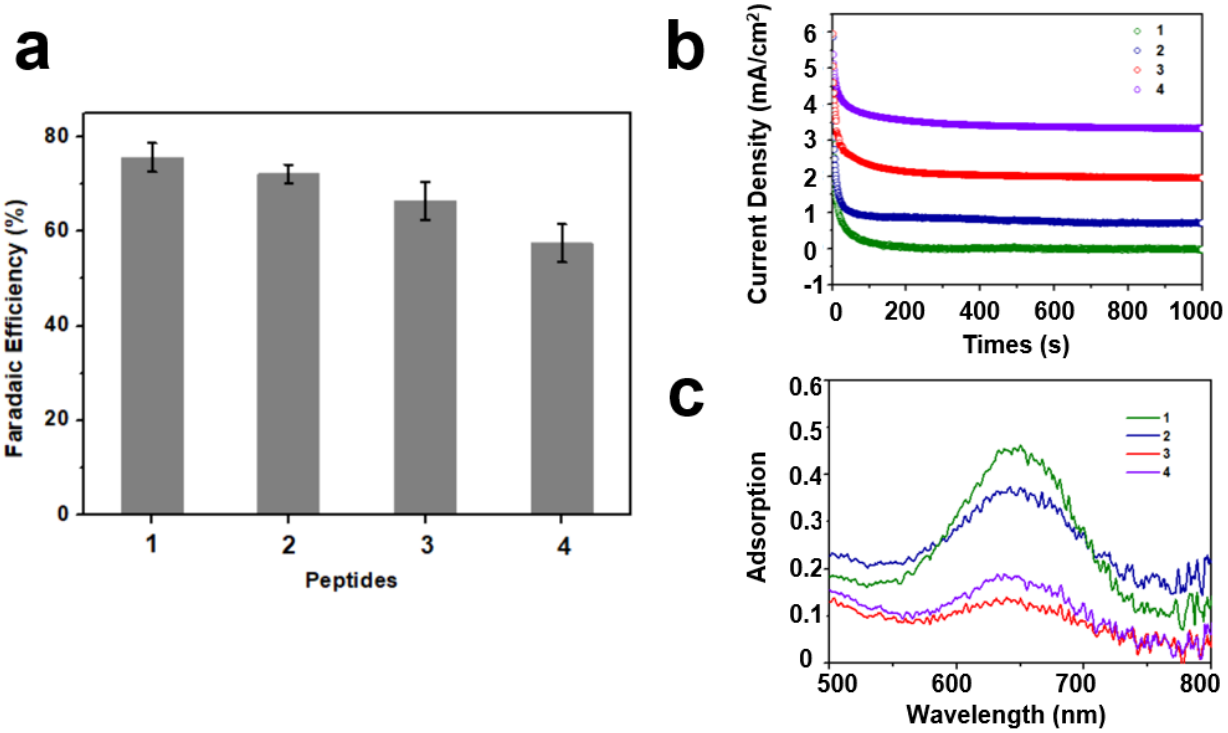

Figure S13. (a) Calculated Faradaic efficiency for peptides 1,2, 3, and 4. (b) Chronoamperometric curves (I vs. t) with each sample measuring peroxidation reactions with 375-μM TMB and 1-mM H_2O_2. (c) UV-Vis spectra of the solutions after the chronoamperometric measurements. The numbers (**1, 2, 3, 4**) indicate the kinds of peptides.

Faradaic efficiency (FE) of electrocatalysis of TMB was calculated using Equation 1.

$$FE = \frac{TMB_{exp}}{TMB_{theo}} \qquad (1)$$

TMB_{exp} is the actual concentration of TMB obtained in the UV-Vis experiment obtained by Equation 2.

$$[c] = \frac{\alpha}{\varepsilon l} \qquad (2),$$

where α is the absorbance of solution after electrocatalytic reaction; ε is the absorption molar extinction coefficient; l is the optical path length.

TMB_{theo} is the concentration of TMB theoretically derived by Equation 3.

$$[c] = \frac{j_{TMB} t}{nFV} \qquad (3),$$

where j_{TMB} is the current density recorded in the chronoamperometry measurements; t is the time in seconds; n is the number of electrons transferred in the reaction; F is the Faraday constant; V is the volume of the solution.

8. Electron transfer performance of the fabricated electrodes.

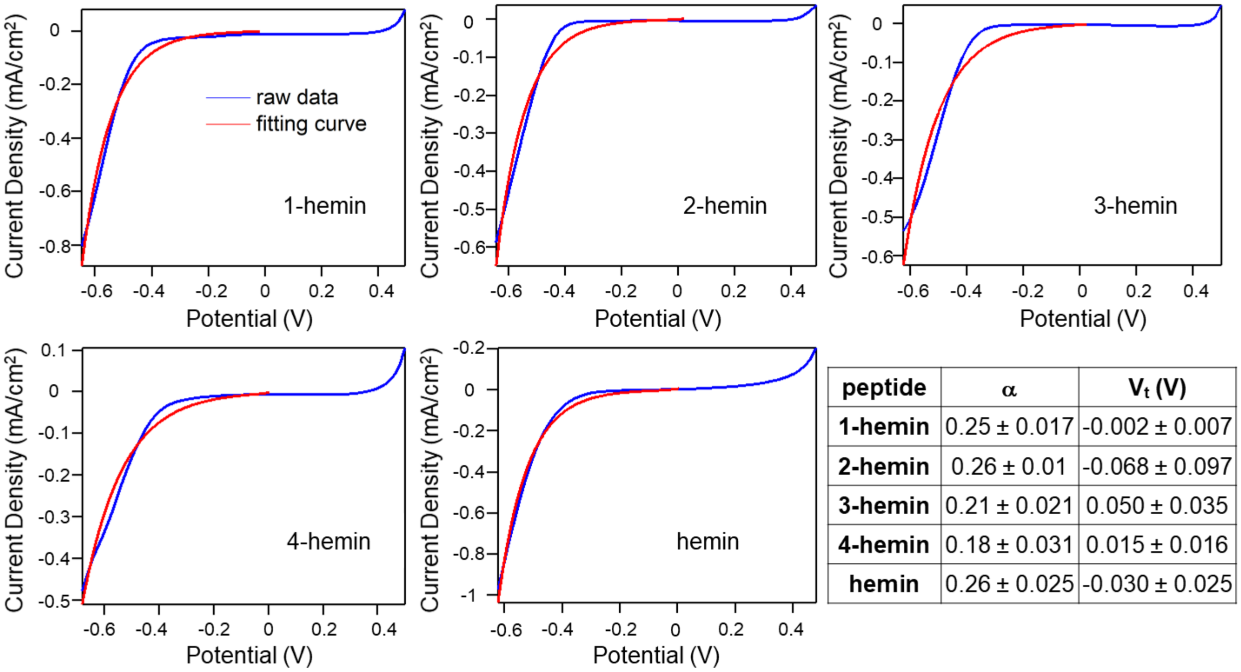

peptide	α	V_t (V)
**1-hemin**	0.25 ± 0.017	-0.002 ± 0.007
**2-hemin**	0.26 ± 0.01	-0.068 ± 0.097
**3-hemin**	0.21 ± 0.021	0.050 ± 0.035
**4-hemin**	0.18 ± 0.031	0.015 ± 0.016
**hemin**	0.26 ± 0.025	-0.030 ± 0.025

Figure S14. Fitting curves (red) of the Butler-Volmer equation with the cyclic voltammogram (blue) of hemin and hemin-peptide electrodes to estimate the electron transfer coefficient α and equilibrium potential V_t.

The electron transfer coefficient α and the equilibrium potential V_t were estimated using the Butler-Volmer equation.

$$j = j_0 \times \{exp\left[\frac{\alpha zF}{RT}(V - V_t)\right] - exp\left[-\frac{(1-\alpha)zF}{RT}(V - V_t)\right]\} \qquad (4),$$

where j is the electrode current density, A/m^2; j_0 is the exchange current density, A/m^2; z is the number of electrons involved in the electrode reaction; F is the Faraday constant; R is the gas constant; T is the absolute temperature (K); V is the electrode potential, V.

Table S3. ΔE_p at different scan rates for each hybrid system.

Scan rate/ V s^{-1}	1-hemin	2-hemin	3-hemin	4-hemin
1	0.228 ± 0.033	0.293 ± 0.025	0.307 ± 0.035	0.311 ± 0.021
0.5	0.214 ± 0.011	0.240 ± 0.017	0.273 ± 0.018	0.262 ± 0.022
0.1	0.196 ± 0.007	0.197 ± 0.010	0.208 ± 0.012	0.225 ± 0.012

Table S4. K_s at different scan rates for each hybrid system.

Scan rate/ V s^{-1}	1-hemin	2-hemin	3-hemin	4-hemin
1	2.44 ± 0.48	1.47 ± 0.24	1.48 ± 0.33	1.52 ± 0.22
0.5	1.36 ± 0.10	1.10 ± 0.13	0.92 ± 0.12	1.01 ± 0.15
0.1	0.33 ± 0.02	0.30 ± 0.02	0.29 ± 0.02	0.28 ± 0.02

The cyclic voltammetry measurements of peptides-hemin hybrid electrodes were carried out at different scan rates (0.1-1 V/s) in the phosphate buffer (0.1 mol/L, pH = 7.0). The heterogeneous electron transfer rates (K_s) was estimated from the peak difference (ΔE_p) and the scan rate (v) by using Laviron's approach,

$$\ln K_s = \alpha \ln(1-\alpha) + (1-\alpha)\ln\alpha - \ln\left(\frac{RT}{nFv}\right) - \alpha(1-\alpha)\frac{nF\Delta E_p}{RT} \quad (5),$$

where α is the electron transfer coefficient; n is the number of electrons transferred in reaction; R is the gas constant; T is the absolute temperature. ΔE_p was derived from the peak positions in the cyclic voltammograms with various scan rates. Based on the fitting with equation 5 (Fig. S14), the values of ΔE_p and K_s for different scan rates were given in Table S3 and S4, respectively.

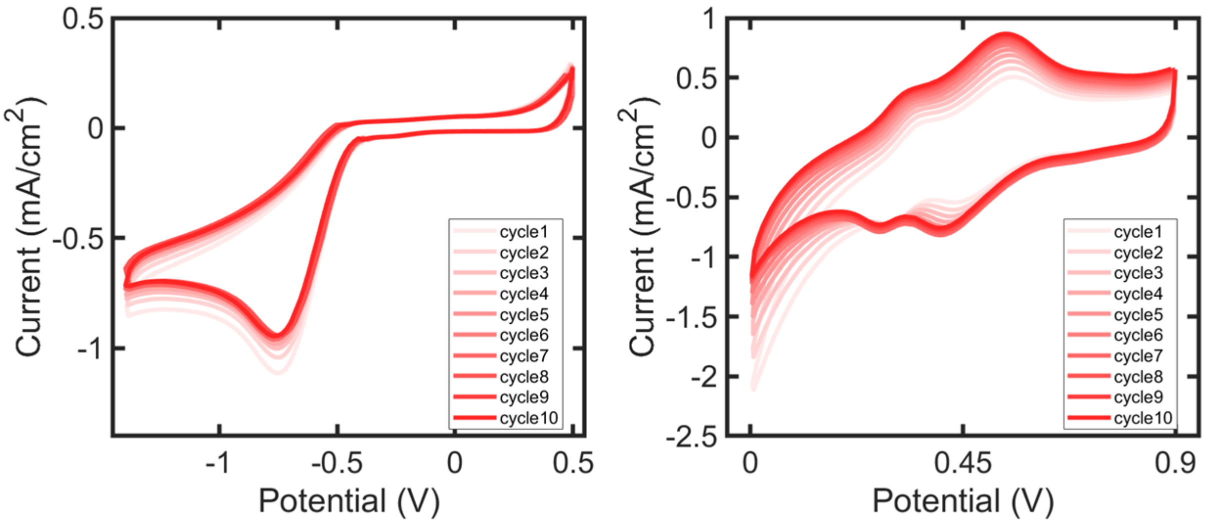

Figure S15. Cyclic voltammograms of hemin-**1**-HOPG electrode with the Ag/AgCl reference electrode (0.198 V vs. Ag/AgCl) and the Pt wire counter electrode at a scan rate of 0.5 V/s. The plots show multiple cycles of the potential sweep to evaluate the stability of the system. (a) H_2O_2 at 1 mM and (b) H_2O_2 at 5 mM and TMB at 100 μM.

REFERENCES

1 Y. H. Christopher R. So Hilal Yazici Carolyn Gresswell Dmitriy Khatayevich Candan Tamerler and Mehmet Sarikaya, *ACS Nano*, 2012, **6**, 1648–1656.

2 T. Xue, S. Jiang, Y. Qu, Q. Su, R. Cheng, S. Dubin, C. Y. Chiu, R. Kaner, Y. Huang and X. Duan, *Angew. Chemie - Int. Ed.*, 2012, **51**, 3822–3825.

3 H. Sun, A. Zhao, N. Gao, K. Li, J. Ren and X. Qu, *Angew Chem Int Ed Engl*, 2015, **54**, 7176–7180.

4 Y. Song, K. Qu, C. Zhao, J. Ren and X. Qu, *Adv Mater*, 2010, **22**, 2206–2210.

5 Y. J. Long, Y. F. Li, Y. Liu, J. J. Zheng, J. Tang and C. Z. Huang, *Chem Commun*, 2011, **47**, 11939–11941.

6 Y. Zhang, C. Wu, X. Zhou, X. Wu, Y. Yang, H. Wu, S. Guo and J. Zhang, *Nanoscale*, 2013, **5**, 1816–1819.

7 R. Li, M. Zhen, M. Guan, D. Chen, G. Zhang, J. Ge, P. Gong, C. Wang and C. Shu, *Biosens Bioelectron*, 2013, **47**, 502–507.

8 S. Zhu, X. E. Zhao, J. You, G. Xu and H. Wang, *Analyst*, 2015, **140**, 6398–6403.

9 L. Gao, J. Zhuang, L. Nie, J. Zhang, Y. Zhang, N. Gu, T. Wang, J. Feng, D. Yang, S. Perrett and X. Yan, *Nat Nanotechnol*, 2007, **2**, 577–583.

10 R. Qu, L. Shen, Z. Chai, C. Jing, Y. Zhang, Y. An and L. Shi, *Acs Appl Mater Interfaces*, 2014, **6**, 19207–19216.

11 F.-X. Qin, S.-Y. Jia, F.-F. Wang, S.-H. Wu, J. Song and Y. Liu, *Catal. Sci. Technol.*, 2013, **3**, 2761.

12 Q. Liu, K. Wan, Y. Shang, Z. G. Wang, Y. Zhang, L. Dai, C. Wang, H. Wang, X. Shi, D. Liu and B. Ding, *Nat Mater*, , DOI:10.1038/s41563-020-00856-6.

13 Y. Zhang, C. Xu and B. Li, *RSC Adv.*, 2013, **3**, 6044.

14 F. Liu, J. He, M. Zeng, J. Hao, Q. Guo, Y. Song and L. Wang, *J. Nanoparticle Res.*, , DOI:10.1007/s11051-016-3416-z.

15 L. Cui, J. Wu, J. Li and H. Ju, *Anal Chem*, 2015, **87**, 10635–10641.

16 L. Wu, G. Wan, N. Hu, Z. He, S. Shi, Y. Suo, K. Wang, X. Xu, Y. Tang and G. Wang, *Nanomater.*, , DOI:10.3390/nano8070451.

17 Vinita, N. R. Nirala, M. Tiwari and R. Prakash, *Mikrochim Acta*, 2018, **185**, 245.

18 J. Sun, C. Li, Y. Qi, S. Guo and X. Liang, *Sensors (Basel)*, , DOI:10.3390/s16040584.

19 V. K. Singh, P. K. Yadav, S. Chandra, D. Bano, M. Talat and S. H. Hasan, *J Mater Chem B*, 2018, **6**, 5256–5268.
